# Inertial cavitation of lyophilized and rehydrated nanoparticles of poly(L-lactic acid) at 835 kHz and 1.8 MPa ultrasound

**DOI:** 10.1038/s41598-019-48074-8

**Published:** 2019-08-21

**Authors:** Pia Hiltl, Alexander Grebner, Michael Fink, Stefan Rupitsch, Helmut Ermert, Geoffrey Lee

**Affiliations:** 1 Division of Pharmaceutics, Department of Chemistry & Pharmacy, Friedrich-Alexander University, Erlangen, Germany; 2 Chair of Sensor Technology, Department of Electrical, Electronic & Communication Engineering (EEI), Friedrich-Alexander University, Erlangen, Germany

**Keywords:** Nanoparticles, Nanoparticles

## Abstract

Nanoparticles of poly-L-lactic acid dispersed in water and of approximately 120 nm diameter were prepared by a nanoprecipitation method followed by lyophilization together with trehalose. After rehydration, the nanodispersion was exposed to ultrasound at 835 kHz frequency and 1.8 MPa peak negative sound pressure. Substantial levels of broadband noise were surprisingly detected which are attributed to the occurance of inertial cavitation of bubbles present in the dispersion. Inertial cavitation encompasses the formation and growth of gas cavities in the rarefaction pressure cycle which collapse in the compression cycle because of the inwardly-acting inertia of the contracting gas-liquid interface. The intensity of this inertial cavitation over 600 s was similar to that produced by Optison microbubbles used as contrast agents for diagnostic ultrasound. Non-lyophilized nanodispersions produced negligible broadband noise showing that lyophilization and rehydration are requirements for broadband activity of the nanoparticles. Photon correlation spectroscopy indicates that the nanoparticles are not highly aggregated in the nanodispersion and this is supported by scanning (SEM) and transmission (TEM) electron micrographs. TEM visualized non-spherical nanoparticles with a degree of irregular, non-smooth surfaces. Although the presence of small aggregates with inter-particulate gas pockets cannot be ruled out, the inertial cavitation activity can be explained by incomplete wetting of the nanoparticle surface during rehydration of the lyophilizate. Nano-scale gas pockets may be trapped in the surface roughness of the nanoparticles and may be released and coalesce to the size required to nucleate inertial cavitation on insonation at 835 kHz/1.8 MPa.

## Introduction

Application of pulsed ultrasound to a pure liquid produces inertial cavitation when gas cavities that are formed and grow in the rarefaction cycle collapse in the compression cycle because of the inwardly-acting inertia of the contracting gas-liquid interface^1^. This leads to acoustic emissions with a wide frequency spectrum, i.e., broadband noise that can readily be detected^2^ and is used as an indicator that inertial cavitation has taken place^3,4^. The inertial cavitation threshold is that peak rarefactional sound pressure necessary to produce a detectable inertial cavitation event. In the medical diagnostic frequency region of ≥500 kHz, the inertial cavitation threshold of pure water is 7 MPa^5^. This threshold can be reduced in the presence of micrometer-sized, stabilized gas bubbles within the water. The reduction in threshold is greater as the size of the dispersed gas bubbles increases^6,7^, i.e. less sound pressure is needed to produce inertial cavitation. This explains the utility of so-called stabilized microbubbles of, for example, 1–10 µm diameter^6^ which reduce the inertial cavitation threshold of pure water to below 1 MPa at ≥500 kHz^7^. Inertial cavitation is also observed in aqueous dispersions of solid microspheres. In one published example the presence of 1 µm diameter polystyrene spheres reduced the inertial cavitation threshold of water to 1.1 MPa at 757 kHz^5^. Both Atchley *et al*.^8^ and Holland & Apfel^5^ suggested that the microspheres trap gas pockets on their surface or within aggregates, or that they stabilize microbubbles present in the dispersion against dissolution. In recent work the nucleation energy barrier model of Zhang *et al*.^9^ predicts that the energy barrier for bubble nucleation on the surface of a particle dispersed in a solvent will be decreased as the bubble/surface contact angle goes up, i.e. as solvent wetting of the particle surface becomes poorer. Bubble nucleation will occur preferentially on a hydrophobic than a hydrophilic surface^10^. If the particle size lies in the nanometer range, however, the inertial cavitation response at frequencies ≥500 kHz weakens greatly. Thus, polystyrene nanospheres of 300 nm diameter showed no inertial cavitation at 500 kHz when insonated at up to 2.5 MPa^11^. Indeed, nanospheres of this size can reduce the inertial cavitation threshold of water only at frequencies below 100 kHz. For example, at 20 kHz polystyrene nanospheres of diameter 280 nm reduced the cavitation threshold of water to 0.34 MPa^6,12^. Silicon dioxide nanospheres of 100 nm diameter also reduced the cavitation threshold of water on insonation at 20 kHz in a concentration-dependent way^13^.

The inertial cavitation of microparticles on insonation at medical diagnostic frequencies of ≥500 kHz (focusable in tissue) has already been exploited to trigger controlled drug release^14,15^. This is also possible with nanoparticles in the upper nanometer range^16^ as has been successfully demonstrated with the hollow polymer ‘nanocup’ of around 500 nm diameter that traps a gas bubble within its single cavity when lyophilized and rehydrated^17^. This entrapped gas pocket is large enough to show inertial cavitation at 500 kHz at 0.5–3 MPa pressure, depending on cavity size^17^. Solid nanospheres cannot, however, entrap individual gas pockets of the size required for inertial cavitation at ≥500 kHz. Kwan *et al*.^18^ solved the Rayleigh-Plesset equation for the surface of a solid nanosphere having a gas-filled surface crevice. A crevice radius of 50–100 nm was required to reduce the inertial cavitation threshold of water to below 1.5 MPa at 500 kHz^18^. Clearly, a solid nanosphere of diameter of about 300 nm could hardly accommodate crevices of this size. Wagstaffe *et al*.^16^ used a layer-by-layer procedure to coat polystyrene nanosphere-cores with colloidal silica nanoparticles. These reduced the inertial cavitation threshold of water down to 0.5 MPa at 1 MHz. It was suggested that the coat could trap very small gas pockets because of incomplete wetting, similar to Atchley *et al*.^8^ and Holland & Apfel^5^. On insonation these emerge during the rarefaction cycle and coalesce to larger bubbles of the size required for inertial cavitation^18^.

This is the starting point of the work presented in this paper. We have achieved the synthesis of simple but cavitable solid nanospheres that are made of a polymer suitable for parenteral application to humans, i.e. polylactic acid, and avoid the use of colloidal silica. An established nanoprecipitation technique^19,20^ was used to produce a dispersion of nanospheres of diameter around 120 nm. These should not lower the inertial cavitation threshold of water at frequencies ≥500 kHz^6,11,12^. Yet when the nanosphere dispersion was lyophilized together with trehalose as a stabilizing^21^ agent (as already used with the nanocups^17^), then on rehydration the nanoparticles were surprisingly quite strongly sonoactive at 835 kHz/1.8 MPa. In this paper we present our results on the characterization of the inertial cavitation activity of these nanospheres. These were prepared not containing any active agent, as the object of the study was to examine the unexpected inertial cavitation activity of the nanostructure *per se*. Because of their simple structure they have the potential for further development as drug-loaded nanocapsules as parenteral drug carrier for ultrasound-induced release.

## Experimental

### Materials

Poly(L-lactic acid) as Resomer L206S (PLA) and D-(+) trehalose dihydrate were obtained from Sigma-Aldrich (Taufkirchen, Germany). Dichloromethane and acetone were obtained from Carl Roth (Karlsruhe, Germany), and Poloxamer 188 as Lutrol F68 was purchased form BASF (Ludwigshafen, Germany). The ultrasound standard was Optison microbubbles (GE Healthcare; Solingen, Germany) which was diluted by adding 59.5 µL to 3.50 mL of a 5% w/v aqueous glucose solution. Polystyrene nanospheres of nominal mean diameter 300 nm and dispersed to 10% w/v in water were purchased from Sigma-Aldrich (Gillingham, UK). Cellulose mixed ester (CME) membrane filters of pore diameter 0.8 µm (Rotilabo, ROTHPU20.1) were obtained from Carl Roth (Karlsruhe, Germany). Water was double-distilled from an all-glass apparatus and then passed through a 0.1 µm pore diameter polyethersulfone membrane filter (Sartorius Stedim, Göttingen, Germany) before use.

### Nanoparticle preparation (nanoprecipitation)

We used the double-syringe impingement technique that we described before^20^ to improve the yield of a nanoprecipitation method^19^. In brief, this ensured effective dispersion of an organic phase solution of PLA in dichloromethane/acetone in an aqueous phase solution containing 0.27% w/v poloxamer 188 and 15% w/v trehalose. This technique allowed multiple passes through the impingement chamber instead of the single pass technique^22^. The weight ratio of PLA/trehalose in the mixed solutions was 1:250 (15 mg polymer and 3.75 g trehalose). The resulting nanodispersion was passed through a 0.8 µm pore diameter membrane filter to remove any large particles that might be present. This preparation is called the *nanodispersion*. 3 mL aliquots were then filled into glass injection vials (10 R) which were placed into a bath of liquid nitrogen for rapid freezing. The frozen nanodispersions in the vials were then transferred to a pre-cooled shelf of a Christ Delta 1–24 KD lyophilizer (Martin Christ, Osterode am Harz, Germany) of shelf area 0.31 m^2^. The lyophilization cycle given in Table 1 is routine for drying aqueous trehalose solutions^23^.

**Table 1:** Lyophilization cycle used to prepare nanoparticles.

Time [hh:mm]	Shelf temperature [°C]	Chamber pressure [mbar]	Phase
00.00	−40	1000	equilibration
02:00	−40	1000	equilibration
02:45	−40	0.1	ramp
04:45	−20	0.1	1° dry
52:45	−20	0.1	ramp
54:25	+20	0.1	ramp
72.45	+20	0.1	2° dry
90:45	+20	0.1	end

The total cycle process time was 72:45 h.

The lyophilized nanosphere dispersions were rehydrated before testing by adding 3 mL water with gentle swirling to yield the *rehydrated nanodispersion*. These were then either examined immediately, or first passed through a 0.8 µm pore-diameter membrane filter. These were examined for acoustic response as described below. Pure water was treated in the same way prior to testing. We made no attempt to measure the colloidal stability of the nanoparticles on storage. This was because each lyophilisate was rehydrated and then used immediately for an insonation experiment which lasted <600 seconds.

### Acoustic response

**Figure 1:**
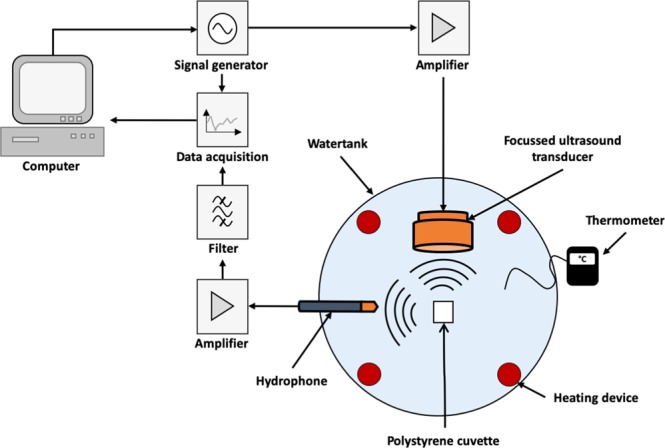
Schematic diagram of ultrasound test device. The focused ultrasound transducer works at 835 kHz and 1.8 MPa pressure. The hydrophone receiver is aligned to the center focus of the cuvette at an angle of 90° to the transmitter and captured ultrasound up to 20 MHz. For further details see text.

The ultrasound test device (Fig. 1) was developed further from our prototype^20^ and was broadly similar to those rigs described by other authors^3,4,16^. It was submerged in an acrylic glass tank filled with deionized, degassed water. This was thermostatted by four heating elements to improve consistency of temperature of the cuvette. Spherically-focussed ultrasound at 835 kHz frequency and with a peak rarefaction pressure of 1.8 MPa was generated from a transmitter comprising an arbitrary function generator (Agilent 33500B Series; Santa Clara, CA-USA) and an RF amplifier model 1160LA. A 4 mL sealed polystyrene cuvette that contained a 3 mL sample of the dispersion being examined was located in the focus region of the transmitter. The transmitter operated in a burst-mode framework with a pulse duration of 1 ms and repetition frequency of 0.5 Hz. The hydrophone receiver (Onda HGL-0200) was aligned to the center focus of the cuvette at an angle of 90° to the transmitter and captured ultrasound up to 20 MHz. This angular misalignment ensured that the hydrophone detected mainly the broadband noise signal generated within the sample. The hydrophone’s output voltage was recorded during the time windows when the focused transducer (Olympus, Europa, Hamburg, Germany; Model V315) was transmitting. After Fourier transformation, its frequency spectrum was analyzed in the region between the sixth and seventh harmonics of the transmitter signal, i.e. 6.0–6.5 MHz, which represents the pure broadband noise caused by cavitation. The voltage spectral density of the hydrophone signal was calculated as we have described before^24^ for each pulse transmit cycle as a measure of the noise level using standard Matlab software. This assumes that the noise level is constant between 6.0 and 6.5 MHz, which is the case. The resulting kinetic trace of the voltage spectral density values versus time reveals the intensity and duration of the broadband noise arising from inertial cavitation^4^ within the sample insonated at 835 kHz/1.8 MPa pulsed ultrasound.

Measurements were performed on a standard that is known to show broadband noise at ≥500 kHz: Optison microbubbles. Two negative controls were also examined: pure water and a non-lyophilized dispersion of polystyrene nanospheres of 300 nm diameter which were known to show no inertial cavitation at this frequency^11^. The subsequent measurements of the aqueous nanosphere dispersions were done on both the non-lyophilized nanodispersion and the rehydrated nanodispersion.

### Photon correlation spectroscopy (PCS)

The particle size intensity distributions of the nanodispersions were determined using a Zetasizer Nano ZS (Malvern Instruments, Malvern, UK) with PS cuvettes. The refractive indices and viscosities of the solvent systems were determined beforehand. Each sample was measured three times, each with a minimum of ten individual runs, and the density (q_3_) and cumulative (Q_3_) distributions determined. From these the value for cumulants Z-average (Z_av_) was calculated. The zeta potential of the dispersed nanoparticles was also determined using the Zetasizer Nano ZS.

### Scanning electron microscopy (SEM) & transmission electron microscopy (TEM)

The nanoparticles were visualized by electron microscopy. SEM was performed on a Carl Zeiss Gemini Ultra 55 machine fitted with a field emission gun run at 100 V-30 kV. It was first necessary to remove the trehalose from the nanodispersions to allow visualization of the isolated nanoparticles in the dried state^25^. For SEM, this was done by dialysis using Spectra/Por 1 mL Float-a-Lyzer tubes with a molecular weight cut-off of 50 kDa floating on 100 mL water. The absence of the trehalose after dialysis was confirmed by a megazyme trehalose assay and also Fourier transformation infra red spectroscopy. A droplet of the dialyzed nanodispersion was then placed on an Al stub and allowed to dry at ambient conditions. This was then Au sputtered (Hummer JR Technics, Munich, Germany) in an argon atmosphere for 5 min at 5 kV/20 mA. Both the non-dialyzed and dialyzed, trehalose-free nanodispersions were examined. TEM was performed on a Philips CM30 TEM/STEM machine using a LaB6 cathode at 300 kV. The trehalose was removed from the nanodispersion for TEM by serial washing. A 2 µL droplet of nanodispersion was placed on a holey carbon copper grid (Plano GmbH, Wetzlar, Germany) and left for 3 minutes before being drawn through the grid by placing a filter paper on the grid’s lower side. A 5 µL droplet of phosphotungstic acid (2% w/w) was then placed on the grid, left for 2 minutes and then drawn through the grid by the same filter paper technique. This was followed by 2 washings with water, again using the filter paper technique. The sample was then examined by the TEM. Both the nanodispersion and a nanoparticle-free dispersion (placebo) were examined.

## Results and Discussion

### Insonation behavior

**Figure 2:**
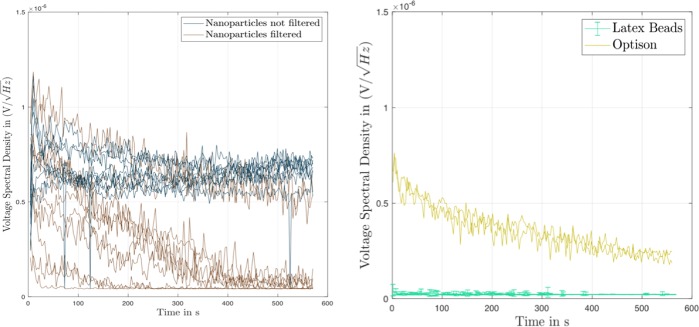
Acoustic response of different nanodispersions on insonation at 835 kHz and 1.8 MPa in the ultrasound test device. The results are given as plots of the voltage spectral density [V/(Hz)^1/2^], versus time [s] for the replicate individual samples. (**a**) Left-hand frame: Rehydrated nanodispersions, either non-filtered (dark blue) or filtered (brown). (**b**) Right-hand frame: Polystyrene nanospheres of diameter 300 nm,10% w/v in water; and Optison microbubbles in water.

**Figure 3:**
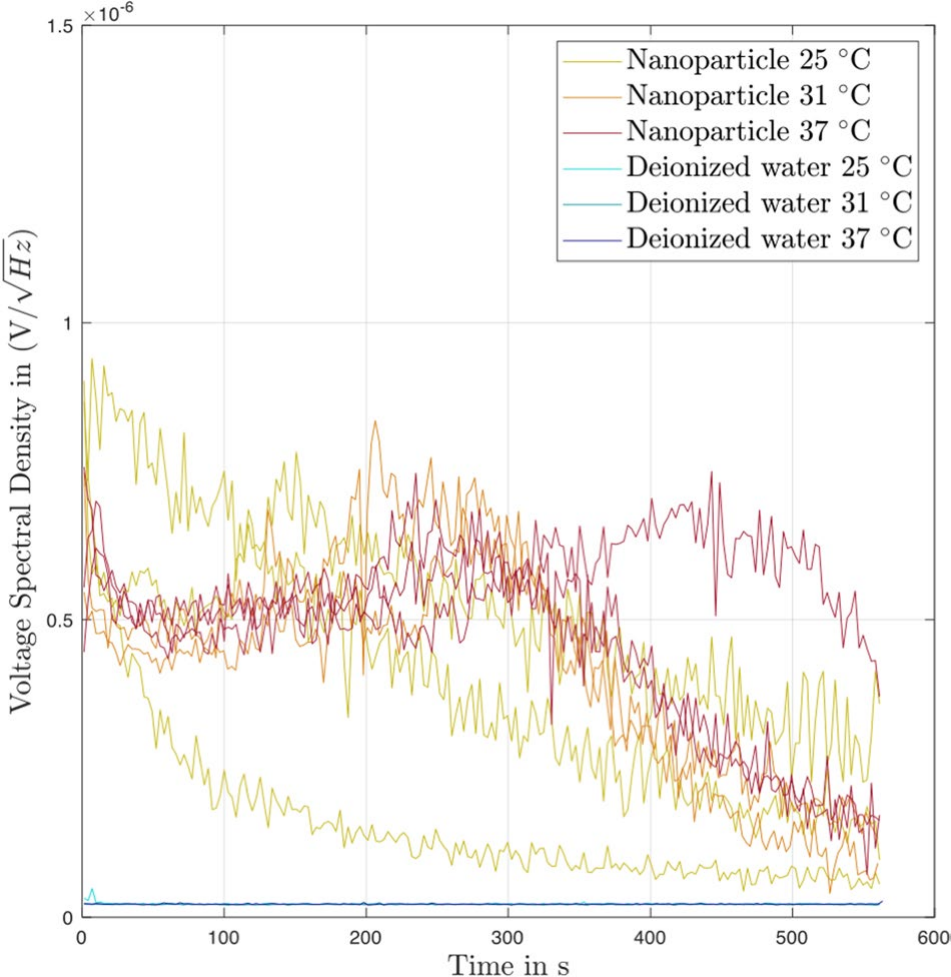
Effect of temperature on acoustic response of nanodispersions on insonation at 835 kHz and 1.8 MPa in the ultrasound test device. The results are given as plots of the voltage spectral density [V/(Hz)^1/2^], versus time [s] for the replicate individual samples. Filtered nanodispersions, and pure water as a negative control.

The upper traces of the voltage spectral density versus time shown in Fig. 2a are for individual samples of the rehydrated, non-filtered nanodispersions. Recall that the insonation of the samples was pulsed with a pulse duration of 1 ms (835 cycles per pulse at 835 kHz) at a repetition frequency of 0.5 Hz. The replicate samples show a consistent pattern of an initial rapid increase in broadband noise during the first approximately 50 s which is followed by a protracted region of a more-or-less constant voltage spectral density of approximately 7 nV/(Hz)^1/2^ over the duration of 570 s of insonation. The variability in sonic response between the replicate samples is quite large, as quantified by the area under the curve, AUC_0→575s_, of voltage spectral density (V/Hz^1/2^) versus time, t, of 3.72 ± 3.41 (n = 9) (Table 2). This quantifies the total sonic response of the dispersion to insonation. The lower traces in Fig. 2a for the samples of rehydrated and filtered nanodispersion also show the initial rapid increase in the voltage spectral density, but this is followed by a continual decline in broadband noise. The rate of decline differs between the individual samples, with one sample falling to zero after 25 s and another showing broadband noise up to 500 s. As a result, the value of AUC_0→575s_ is halved (Table 2). The non-lyophilized nanodispersion showed only a negligible level of broadband noise (graph not shown) and behaved as pure water shown as the lower profile in Fig. 3. The measured voltage spectral density was negligible and ran at a level of below 1 nV/(Hz)^1/2^ over the duration of insonation with an AUC_0→575s_ of just one hundredth of that of the filtered nanodispersions (Table 2). It follows that the levels of broadband noise measured in Fig. 2a are caused in some way by the lyophilization and rehydration treatments of the nanodispersions. In addition, filtering the rehydrated nanodispersions through a 0.8 µm pore diameter membrane filter weakens the broadband response (see AUC_0→575s_ in Table 2).

**Table 2:** Quantification of area under curve, AUC_0→575s_, of voltage spectral density (V/Hz^1/2^) versus time, t, for various dispersions treated for 575 s at 835 kHz/1.8 MPa.

Preparation	AUC_0→575s_, [V s/Hz^1/2^] × 10^−3^ mean average ± SD	Figure
Rehydrated nanodispersion, non filtered	3.72 ± 3.41 (n = 9)	2a
Rehydrated nanodispersion, filtered	1.62 ± 1.30 (n = 10)	2a
Polystyrene nanoparticles	0.0132 ± 0.0014 (n = 5)	2b
26 °C: Optison	0.20 ± 0.011 (n = 3)	2b
26 °C: rehydrated nanodispersion, filtered	0.20 ± 0.098 (n = 3)	3
31 °C: rehydrated nanodispersion, filtered	0.24 ± 0.012 (n = 3)	3
37 °C: Rehydrated nanodispersion, filtered	0.27 ± 0.042 (n = 3)	3
Water 25 °C	0.012 ± 0.0002 (n = 3)	3

Broadband noise is generated by the inertial cavitation of gas bubbles^26^. The resulting shock waves emitted by the collapsing bubbles produce the broadband noise^4^ whose level can be used as a quantitative measure of inertial cavitation^3,4^. The lyophilization and rehydration treatments of the nanodispersion evidently lead to the ability to show inertial cavitation activity at 835 kHz/1.8 MPa. Compare this behavior with that of the dispersion of polystyrene nanospheres of Z_av_ = 300 nm in Fig. 2b which had been lyophilized with the same amount of trehalose at the same volume fraction of particulate material. This shows negligible activity (≤0.2 nV/(Hz)^1/2^) over 550 s which is the same as that seen with pure water as the lower profile in Fig. 3. We conclude that the rehydrated nanoparticles can act as nucleation sites for inertial cavitation in the dispersion at 835 kHz/1.8 MPa. The acoustic response is, however, unexpected since the polystyrene nanospheres show no activity (cf. Fig. 2b) and it has been reported that polystyrene nanospheres of this size did not reduce the cavitation threshold of pure water at 500 kHz/1.5 MPa^6,11^.

The relative strength of the inertial cavitation of the rehydrated nanoparticles can be assessed by comparing their broadband emissions with those of an Optison standard. The upper traces in Fig. 2b are the individual results of voltage spectral density versus time for Optison microbubbles (albumin-stabilized perflutren^27^). This should be compared with Fig. 3 which shows the kinetic traces of voltage spectral density for filtered nanoparticles from the same experiment at three different temperatures. These results show two issues of interest. First, the Optison microbubbles in Fig. 2b and the filtered nanoparticles in Fig. 3 produce broadly similar levels of broadband noise. At 26 °C, the values for the voltage spectral density with both dispersions start at approximately 7 nV/(Hz)^1/2^ and decline to 4 nV/(Hz)^1/2^ after 200 s. Additionally, the values of AUC_0→575s_ are similar for Optison and the filtered nanoparticles at this temperature (Table 2). This suggests that Optison and the filtered, rehydrated nanoparticles enclose similar amounts of gas bubbles. It has been shown that the level of broadband noise depends on the amount, i.e. volume, of gas set into inertial cavitation by a sound pressure wave^4^. The volume fraction of gas, *ϕ*_gas_, present in the diluted Optison at the start of insonation in our experiments can be estimated from the number of bubbles per mL (6.5 × 10^8^ in pure Optison^3^) and taking an average bubble diameter of 5 µm^27^. The result is *ϕ*_gas_ ∼ 7.2 × 10^−4^. The amount of gas trapped by the rehydrated PLA nanospheres is unknown; only the volume fraction of the solid polymer of the nanoparticles, 𝜙_v_, is known and is ∼4.6 × 10^−4^ (assuming a 100% yield from nanoprecipitation). The similar acoustic response indicates that even the filtered, rehydrated nanodispersions must have an amount of enclosed gas equal to or larger than the amount of solid nanoparticles present. This could be the case if the nanoparticles were either aggregated to form inter-particulate gas pockets, or if the nanoparticle surface was incompletely wetted allowing gas bubbles to be trapped on the surface of monodispersed nanoparticles, two of Holland & Apfel’s^5^ suggestions.

Secondly, the kinetic profiles of the voltage spectral density of the filtered nanoparticles (Fig. 3) show a marked temperature-dependent response. Despite the visibly obvious variation in the replicates (n = 3 at each temperature), this temperature-dependence is observed starting after approximately 100 s when the individual profiles for the different temperatures start to diverge. The degree of broadband noise becomes higher on moving from 25 °C through 31 °C and onto 37 °C which gives the highest response of all 3 temperatures, i.e., the sequence 25 °C < 31 °C < 37 °C. The values for mean average AUC_0→575s_ in Table 2 increase at higher temperature. Webb *et al*.^28^ solved the Keller-Miksis equation to show that increase in temperature causes the cavitation threshold of water to drop. In the nanometer range of size of gas bubble–which should be relevant for the filtered nanoparticles–the cavitation threshold was predicted by the model to decrease linearly with temperature from 20 °C to 80 °C. This increase in inertial cavitation activity with temperature depended on the effects of temperature on surface tension, viscosity and vapor pressure. In Fig. 3 the kinetic profiles of the voltage spectral density measured at 25 °C show a continued decline with time, whereas at the two higher temperatures the profiles run through an initial decrease, followed by an increase to a peak value before declining to the end of the measurement.

We note that the acoustic behavior of Optison in Fig. 2b differs from that of a previous study with Optison insonated at 500 kHz and similar pressure, P, where a peak in broadband noise occurred at less than 0.5 s after the start of insonation followed by a rapid decline over just 2 s^3^. This much shorter duration of broadband noise than seen in the current work may have been a result of the approximately forty-times longer pulse length of t = 60 ms at 1 Hz used than the t = 1 ms at 0.5 Hz used in our study. The ultrasound energy exposure of the focal point in the dispersion, E, will therefore be approximately forty-times higher, since E = P^2^t/𝜌c where 𝜌 is the water density and c is the speed of sound through water^3^. Again, we assume that also with Optison the broadband noise comes from inertial cavitation^26^, in this case of the microbubbles in Optison.

### Nanoparticle morphology

**Figure 4:**
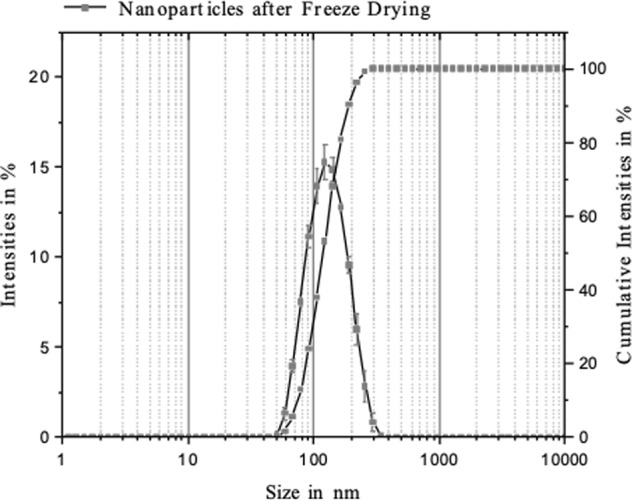
Results of dynamic light scattering (PCS) measurements of rehydrated nanodispersion. Z_av_ = 122 nm. The q_3_ and Q3 particle size distributions are shown.

The nanodispersions when examined before insonation were slightly turbid as a result of the low volume fraction of the precipitated polymer of the nanoparticles, 𝜙_v_, of ∼4.6 × 10^−4^. The q_3_ (density) and Q_3_ (cumulative) particle size distributions of the nanodispersion before lyophilization give a Z-average diameter, Z_av_, (see Table 3) of 110 ± 1 nm (n = 4). These are the nanoparticles produced by the double-syringe impingement technique and are dispersed in the aqueous 15% w/v trehalose solution. These nanoparticles are therefore much smaller than the approximately 230 nm given by the original nanoprecipitation method^19^ which used a less intensive mixing of organic and aqueous phases during polymer precipitation. After lyophilization and rehydration, the q_3_ and Q_3_ distributions shifted visibly to larger sizes and the Z-average diameter increased slightly to 122 ± 1 nm (Table 3). This reflects the increase in the width of the distribution (best seen in q_3_) from 40–103 nm before lyophilization to 40 nm - 135 nm after lyophilization and rehydration (Fig. 4). This could be because some small aggregates were formed on lyophilization which did not disaggregate on rehydration, or the presence of gas pockets attached to the surface which move with the nanoparticle. After insonation of this nanodispersion for 600 s at 835 kHz/1.8 MP, the Z_av_ fell to 119 ± 1 nm (Table 3) and the distribution width was observed to have been reduced to 40 nm–120 nm (not shown). This could have been caused by some disaggregation of the nanoparticles, or the loss of gas pockets from the nanoparticle surface on insonation.

**Table 3:** Results of photon correlation spectroscopy (PCS) measurements of nanoparticles before lyophilization, after lyophilization and rehydration, and after ultrasound treatment of 600 s at 835 kHz/1.8 MP.

Dispersion	Z Average size, Z_av_ [nm]	Quotient Z_av_^before^/Z_av_^after^	PDI
Before lyophilization	110 ± 1.0	1.00	0.07 ± 0.014
After lyophilization and rehydration	122 ± 1.9	1.14	0.11 ± 0.009
After ultrasound of rehydrated lyophilisate	119 ± 1.2	1.08	0.15 ± 0.023

Each result is the mean average ± standard deviation (n = 4).

**Figure 5:**
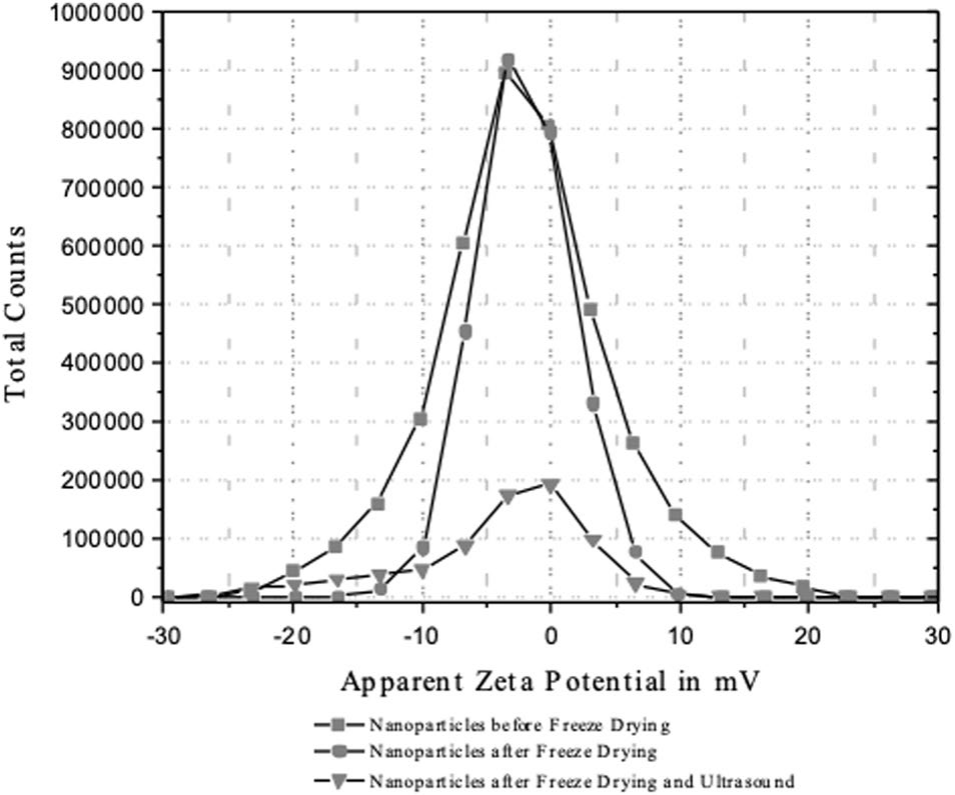
Results of zeta potential determinations of nanodispersions. The different nanodispersions are: non-lyophilized nanodispersion; rehydrated nanodispersion; rehydrated nanodispersion after 600 s insonation at 835 kHz/1.8 MPa.

The effect of lyophilization and rehydration on the size of the nanoparticles can also be expressed as the quotient of Z-average diameters–a routine method^21^. The result is 1.14 which is a little lower than that of 1.20 reported for lyophilized PLA-nanoparticles of 200 nm initial diameter at a PLA/trehalose weight ratio of 1:8^21^. In the current work, we used a much higher weight ratio of PLA/trehalose of 1:250. It has, however, been shown that the stabilization of nanoparticles during lyophilization by disaccharides such as trehalose did not correlate with glass formation to suppress mobility and hence aggregation of the colloidal particles^29^. It appeared that stabilization was more dependent on use of polymeric steric stabilizers such as Poloxamer 338 or Cremophore EL which correlated with reduced aggregation during lyophilization and rehydration^30^. The small changes in Z_av_ and the width of distribution on lyophilization and rehydration seen in our work may therefore be related–at least in part–to the long-chain, polymeric surfactant used here, Lutrol F 68, of molecular weight 7,680–9,510 and comprising approximately 79 EtO units in each of two chains^31^. This idea is supported by the result for zeta potential of the nanoparticles which in each nanodispersion (non-lyophilized; lyophilized and rehydrated; insonated) shows a wide distribution of values between −30 mV and +20 mV (Fig. 5). The mean average values are slightly negative, being −2.1 mV, −1.9 and −4.1 mV, respectively. These values are much lower than the 30–60 mV considered to be necessary for good colloidal stability in dispersion in the absence of polymeric steric stabilization^32,33^. The explanation of this is that a low zeta potential of nanoparticles can result from the adsorbed layers of long-chain, non-ionic, steric stabilizer which shift the plane of shear further outwards from the nanoparticle surface^34^.

**Figure 6:**
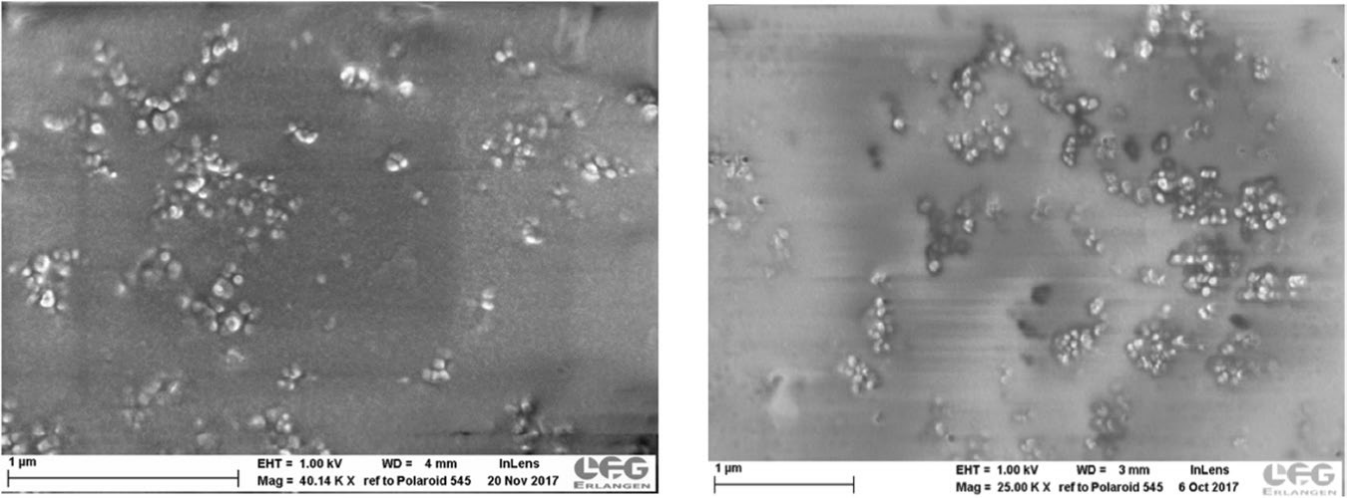
Scanning electron micrographs (SEC) of rehydrated nanodispersions. (**a**) Left-hand frame: non-dialyzed. (**b**) Right-hand frame: dialyzed.

Both the SEM and TEM images give low resolution of the structure of these organic nanoparticles and certainly cannot be compared with those of nanoparticles of inorganic materials^9^. Despite this limitation, the representative SEM image of the non-dialyzed, dried nanodispersion shown in Fig. 6a reveals extensive aggregates of individual nanoparticles which have diameters of between approximately 50 and 100 nm. This result agrees with that of PCS where the particle size distribution in the nanodispersion ranged from 40 nm–103 nm with a Z_av_, of 110 nm (Table 3). The aggregates were therefore not present in the nanodispersion but were formed during the drying of the droplet of nanodispersion placed on the Al stub prior to sputtering. As the dispersion medium is lost via evaporation, the nanoparticles aggregate as the Laplace capillary pressure across the funicular liquid-bridges between the nanoparticles increases^35^. The appearance under SEM is unchanged after dialysis (Fig. 4b).

**Figure 7:**
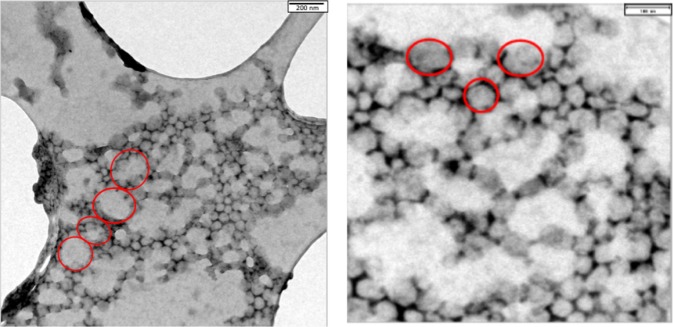
Transmission electron micrographs (TEM) of rehydrated nanodispersion. (**a**) Left-hand frame: Magnification = 12,000x. (**b**) Right-hand frame: Magnification = 24,000x.

The use of TEM resolves somewhat better the surface structure of the nanoparticles since its resolution is about an order of magnitude higher than SEM^36^. Figure 7a is a representative image of the rinsed nanodispersion on the carbon grid. The nanoparticles can be seen on the grid surface and between the grid’s large, empty lacunae. The diffuse, white, cloud-like structures come from the Poloxamer which was evidently not completely rinsed off the grid by the washing procedure. A nanoparticle-free placebo sample had the same appearance (not shown). The higher magnification in Fig. 7a reveals two structural features of the nanoparticles. First, the size range of the individual nanoparticles is approximately 50–100 nm which agrees with both the PCS and SEM results. Bear in mind that PCS determines a hydrodynamic diameter whereas the SEM micrograph yields a projection diameter. The aggregated appearance in Fig. 7a,b was therefore not present in the nanodispersion but is a result of the washing and drying procedure on the carbon grid. Secondly, the nanoparticles have an irregular, non-spherical geometry with surfaces that are not smooth. They have a more irregular surface morphology than nanoparticles of PLA prepared by nanoprecipitation with mechanical stirring^19^ or of PLGA nanoparticles made by ultrasonic dispersion, both of which were near spherical and smooth^37^.

### Origin of broadband noise

The broadband noise measured in our set-up is attributed to inertial cavitation of gas bubbles present in the nanodispersion^4,26^. This inertial cavitation activity occurred only in the rehydrated nanodispersion and not in the non-lyophilized nanodispersion. Nanoparticles of diameter well below 500 nm should not, however, show inertial cavitation at ≥500 kHz^6,10^, as indeed observed in this work with the polystyrene latices and the non-lyophilized nanodispersion. Possible explanations of this behavior are given in the early work published by Atchley *et al*.^8^ who detected a reduction of the cavitation threshold of water by 1 µm diameter microparticles when insonated at 1 MHz. They suggested three possible mechanisms: first, the particles act to stabilize microbubbles present in the dispersion by adsorption at the bubble/liquid interface; secondly, the particles trap nano-sized gas pockets within the inter-particulate spaces of aggregates; thirdly, the particles trap nano-sized gas pockets in the roughness of their surfaces^8^.

The first mechanism does not appear to be likely when viewing our results. The PCS results showed no micrometer-sized, stabilized microbubbles in the rehydrated nanodispersion. It is known that PCS is able to detect the presence of microbubbles or nanobubbles dispersed in water, should they exist^38^. The second mechanism is feasible; although the SEM/TEM results detected no visible aggregates, the SEC distribution gives the presence of particle diameters of up to 350 nm for the rehydrated nanodispersions (Fig. 4) of Z-average 122 nm (Table 3). This size range may be only monomers, but does also encompass the diameter of small aggregates. Aggregated hard spheres of individual diameter D_0_ have theoretical hydrodynamic diameters of 1.20 D_0_, 1.45 D_0_ and 1.48 D_0_ for dimers, trimers and tetramers, respectively^39^. Any small aggregates present could therefore create nano-sized gas pockets trapped within their inter-particulate spaces. This mechanism cannot be excluded, despite the SEM/TEM results. The third possible mechanism is also feasible of nano-sized gas pockets trapped on of the surface of the nanoparticles because of incomplete wetting on rehydration. The result to support this proposition is the TEM images which show that the rehydrated nanoparticles appear to have non-smooth, irregular surfaces which could trap gas pockets in this way. Zhang *et al*.^9^ showed that bubble nucleation on the surface of a particle in dispersion becomes energetically more favorable as the bubble/surface contact angle goes up, i.e., the surface is poorly wetted. This could also explain the weakened broadband noise after filtration of the rehydrated nanodispersion seen in Fig. 2a. This was a pressure filtration process with higher pressure on the apical than on the basal side of the membrane filter. Henry’s law predicts for this case a lower gas solubility in the water emerging from the basal side and possibly better wetting and fewer gas pockets for inertial cavitation whose intensity falls off rapidly with time. We did not, however, attempt to measure the dissolved gas concentration in this study. The loss of nanoparticles during passage of the rehydrated nanodispersion through a 0.8 µm pore diameter filter appears unlikely, even if some had been aggregated. Recall that the Z-average diameter was 122 nm, and the largest detected particles in the cumulants size distributions were at approximately 350 nm (cf. Fig. 4).

For nanoparticles, the Rayleigh-Plesset equation predicts that inertial cavitation can occur at 500 kHz and insonation pressures below 2 MPa provided that surface roughness is sufficient to produce the existence of gas pockets of radius of 50–100 nm^18^. Although this is implausible for our rehydrated nanoparticles of Z_av_ of 122 nm, Kwan *et al*.^18^ proposed an alternative mechanism. These authors calculated that surface-trapped gas pockets much smaller than that predicted by Rayleigh-Plesset may be released from the surface on insonation during the rarefaction cycle and coalesce to larger bubbles of the size required for inertial cavitation.

## Conclusions

Nanoparticles of diameter around 120 nm should not show inertial cavitation at ≥500 kHz^6,10^. This was indeed the case with both the non-lyophilized nanodispersion and the latex dispersion examined here. The novelty of the work presented here is that rehydrated nanoparticles of Z_av_ 122 nm show inertial cavitation at ≥500 kHz which is of similar intensity to that produced by Optison microbubbles. This unexpected behavior must originate in the lyophilization/rehydration treatment of the nanodispersion. The combined results of PCS and SEM/TEM indicate that the nanoparticles are not strongly aggregated in the nanodispersion. The source of the cavitation response could be the non-spherical, non-smooth surface morphology of the nanoparticles as visualized with TEM, although cavitation of small aggregates which create nano-sized gas pockets trapped within the inter-particulate spaces cannot be ruled out. The cavitatable nanospheres have a simple structure that offers the possible combination of cavitation agent with a drug vector in one structure. This would avoid complex structures such as layer-by-layer coated nanosphere-cores or the use of structurally separate cavitation agent and drug vector^40,41^.
